# Beyond Legal Rights: Understanding Mental Health and Autonomy in Criminal Self‐Representation

**DOI:** 10.1002/bsl.2724

**Published:** 2025-03-28

**Authors:** Shai Farber

**Affiliations:** ^1^ Department of Criminology The Max Stern Emek Jezreel Academic College Afula Israel

**Keywords:** autonomy, defendant experiences, defendant motivations, mental capacity and waiver of counsel, pro se defense, self‐representation, vulnerable defendants

## Abstract

This qualitative study examines criminal defendants who waived legal representation to self‐represent in court. Through interviews with 16 participants and courtroom observations, findings reveal intersecting factors driving this decision: mental health challenges, desire for autonomy, attorney mistrust, dissatisfaction with past legal experiences, and underestimation of legal complexities. The research highlights defendants' vulnerability when exercising this right and connects negative prior legal encounters with self‐representation choices. These insights into Israeli pro se defense suggest policy reforms balancing autonomy with support mechanisms. By prioritizing defendants' narratives, this research illuminates self‐representation's social and psychological dimensions, advancing discourse on this understudied phenomenon.

## 1 Introduction

The right to self‐representation in criminal trials is a complex legal and social phenomenon that intersects with autonomy, access to justice, and procedural fairness issues. While legal systems worldwide differ in their self‐representation approaches, concerns persist regarding its implications for defendants' rights and the integrity of judicial proceedings. In jurisdictions such as France and Germany, defendants in severe criminal cases are required to be represented by legal counsel to prevent unfair trials and ensure that procedural rules are properly followed (Bohlander 2008; Hodgson 2005).

In contrast, countries like the U.S.A, England, Canada, and Israel recognize the right to self‐representation but impose safeguards to mitigate its risks (Assy 2015; Farber 2020). These varied approaches reflect fundamental tensions between procedural efficiency and defendant autonomy. The question of whether defendants truly *choose* to represent themselves or whether systemic failures push them toward this option remains a critical issue in legal scholarship.

Despite its firm legal and philosophical foundations, self‐representation's practical impact from the defendant's perspective remains underexplored. Prior studies have largely focused on procedural inefficiencies and case outcomes (Engler 1998; Buhai 2008), while fewer works have examined the motivations, challenges, and lived experiences of self‐represented defendants (Casper 1972; Hashimoto 2010). Research indicates that self‐representation is not always an informed assertion of autonomy; instead, it can reflect a deeper structural issue of legal disenfranchisement. Clair (2021) has demonstrated how defendants' legal choices are shaped by their social position, institutional distrust, and systemic disadvantages.

Similarly, Hashimoto (2010) argues that defendants who self‐represent are often marginalized individuals who feel alienated by traditional legal representation. This study builds upon these insights by examining self‐representation within Israel's criminal justice system—a context where access to legal counsel is relatively accessible through a comparatively liberal public defense policy, yet some defendants still choose to partially or fully forgo this representation despite its availability. The findings offer a deeper understanding of whether self‐representation is a strategic choice, a necessity imposed by systemic distrust, or a response to prior negative legal encounters.

This research engages critical perspectives on self‐representation, addressing structural inequalities in the legal system beyond procedural concerns. Casper (1972) and Clair (2021) argue that self‐representation reflects power imbalances between defendants and the state rather than autonomous choice. Drawing on Foucault's (1975) concept of disciplinary power and Kohler‐Hausmann's (2018) work on misdemeanor justice, this study examines whether self‐representation constitutes an assertion of agency or a response to systemic failures in the legal system.

This qualitative study draws on semi‐structured interviews with 16 purposively sampled self‐represented defendants in Israel, supplemented by courtroom observations. These primary questions guide the research:

1. What motivates defendants to self‐represent rather than use free legal services?
2. What challenges do self‐represented defendants face during trial?

Findings from this study provide empirical insights into the social and psychological dimensions of self‐representation, shedding light on the vulnerabilities of defendants who navigate the legal system without professional assistance. This research has significant legal and policy implications, informing discussions on how courts balance defendant autonomy with the need for procedural fairness. Ultimately, this study contributes to broader debates on human rights, social justice, and access to legal representation, offering a nuanced understanding of how self‐representation operates in practice.

## 2 Literature Review

### 2.1 An International Analysis of Pro Se Defense in Criminal Law

To further investigate the key factors behind the decision to self‐represent, this literature review examines prior research on (1) international approaches to self‐representation, (2) quantitative data on the prevalence of pro se defense, and (3) factors motivating defendants to decline legal representation. Comparing findings across these areas elucidates the need for updated, qualitative empirical work centered on defendants' direct experiences in Israel as a case study.

### 2.2 Approaches to Self‐Representation

While guaranteeing a right to counsel in criminal cases, the U.S. Constitution's Sixth Amendment does not prevent this right from being waived. This allows defendants to decide whether they wish to represent themselves, as established in Faretta v. California (1975). In other countries outside the US, the right to self‐representation may be more limited or conditional, depending on the legal system and case circumstances. For example, the common law and the Criminal Procedure Rules in England and Wales formally recognize the right to self‐representation. However, this right, reluctantly granted, is subject to the discretion of the judge, who may appoint a lawyer to assist or represent the defendant if they consider it necessary in the interests of justice. The judge may also limit the scope of self‐representation (Assy 2015).

In some countries, the right to self‐representation may be explicitly prohibited or discouraged, especially in severe or complex cases or when the defendant is mentally ill or incompetent. For example, in Germany, the law does not recognize the right to self‐representation, and a lawyer must represent the defendant in all criminal cases except for minor offenses. The rationale for this rule is to ensure the fairness and efficiency of the trial and to protect the defendant from self‐incrimination or self‐harm (Bohlander 2008; Van Kalmthout et al. 2009). A similar rule applies in France, where the law requires that a lawyer must assist the defendant in all criminal cases, allowing only a few exceptions. The lawyer may be appointed by the court or chosen by the defendant, but the defendant cannot refuse the lawyer's assistance. The rationale for this rule emphasizes the need to balance the power of the prosecution and the judge (Hodgson 2005).

The right to self‐representation is also recognized in various international and regional human rights rules, such as the Universal Declaration of Human Rights (1948, art. 10), the International Covenant on Civil and Political Rights (1966, art. 14(3)), the European Convention for the Protection of Human Rights and Fundamental Freedoms (1950, art. 6(3)), and the American Convention on Human Rights (1969, art. 8(2)). However, the interpretation and implementation of this right may differ across countries and international jurisdictions, depending on their legal traditions, constitutional provisions, and judicial practices.

The International Criminal Tribunal for the Former Yugoslavia (ICTY) permitted self‐representation with standby counsel support in landmark cases like Prosecutor v. Seselj (2003) and Prosecutor v. Karadžić (2010) (Zahar 2008, 242; Jørgensen 2006; Scharf 2006, 44). Similarly, national systems have developed standby counsel frameworks as procedural safeguards. The U.S. Supreme Court in McKaskle v. Wiggins and 465 U.S. 168 (1984) established that standby counsel may provide guidance without interfering with defendants' control over their defense. Research suggests well‐implemented standby counsel systems balance procedural efficiency with defendant autonomy (Pearson 1984). Despite these international precedents, Israel lacks a formal standby counsel framework despite growing practical concerns about self‐representation.

### 2.3 The Use of the Right to Self‐Representation

The right to self‐representation varies depending on the type and level of the court, the nature and severity of the charges, the availability and quality of legal aid, and the characteristics and preferences of the defendants. There is an ongoing lack of reliable data on the prevalence and patterns of self‐representation across countries and jurisdictions, but some studies have attempted to provide estimates and insights.

The rate of self‐representation in criminal cases in the US is estimated to range from 0.5% to 2.5% of all criminal cases. The resulting percentage varies based on factors such as the stage of the proceedings, whether it is a pre‐trial procedure or a plea agreement, the type of criminal procedure, the sample size, and more. For example, Hashimoto (2010) noted that the number of criminal defendants who chose to proceed *pro se* is “between 0.3% and 0.5%” (p. 1183), while Goldschmidt and Stemen (2015) presented different findings based on another set of parameters.

The right to self‐representation is more commonly exercised in lower courts, such as municipal or district courts, and less so in higher courts, such as state or federal courts. The right to self‐representation is also more common in relatively minor cases, such as misdemeanors or infractions, than in more severe cases, such as felonies or capital offenses (Goldschmidt and Stemen 2015). The right to self‐representation is also influenced by the availability and quality of legal aid, which may vary across states and counties.

Outside the US, the exercise of the right to self‐representation in criminal proceedings may be even less common due to legal or practical constraints and disincentives. For example, in England and Wales, self‐representation is estimated to be used in less than 1% of all criminal cases, and it is mainly confined to minor offenses in the magistrates' courts. However, other estimations suggest that around 20% of defendants are unrepresented for at least one hearing (Kemp 2010; Ministry of Justice and Legal Aid Agency 2015; Welsh 2016). In many countries, such as Israel, where self‐representation has only recently gained semi‐formal recognition through judicial decisions, there are no official statistics on the number of defendants who opt to self‐represent (Farber 2020; Assy 2024).

Self‐representation is significantly more common in civil proceedings (such as family cases, housing disputes, and tort claims) than in criminal proceedings. For example, the USA's Civil Justice Initiative (2015) estimated that 76% of civil cases included self‐represented litigants. In England and Wales, 80% of all family cases in the first quarter of 2014 included self‐represented litigants. A report by the judiciary to the British House of Commons indicates that in the last decade, there was a significant increase in the rates of self‐representation in family proceedings (about 64% in 2017–2018) (Ministry of Justice and Legal Aid Agency 2015; Neill 2019). In Canada, in 2011, 21% of litigants in civil proceedings before the Supreme Court of British Columbia represented themselves (Macfarlane 2013, 26). In Australia, 27% of cases brought before family courts between 2011–2012 involved self‐represented litigants (Faulks 2013, 5).

Judges tend to discourage the use of the right to self‐representation (Goldschmidt et al. 1998). They prefer having a lawyer representing the defendant or, at the very least, offer assistance as needed. They may also limit the scope of self‐representation if they consider it necessary or appropriate. The availability of the legal aid system, which provides free or subsidized legal assistance to defendants based on certain criteria, such as their income and the case's merits, also affects the rate of self‐representation (Goldschmidt 2022). Additionally, criminal procedure's complexity and adversarial nature pose significant challenges and disadvantages for unrepresented defendants. Hence, criminal defendants are often reluctant to rely on self‐representation (Goldschmidt 2022).

A wide array of studies has been conducted to empirically investigate the correlation between the absence of legal representation and the capacity to safeguard legal rights across various legal contexts (Poppe and Rachlinski 2015; Greiner and Pattanayak 2011). For example, Seron et al. 's (2001) study on poor tenant cases in New York City housing courts found that representation decreased eviction rates by 24%. The majority of existing research has demonstrated a positive correlation between legal representation and improved outcomes for litigants (Williams 2011; Poppe and Rachlinski 2015; Greiner and Pattanayak 2011).

Some studies have shown the difference in outcome between self‐represented defendants and represented defendants to be negligible. Still, others have found no correlation at all, and a few have even found a negative impact on defendants resulting from legal representation. Hashimoto (2006), for example, found no difference in the conviction rates of self‐represented defendants compared to represented defendants in criminal proceedings in US state and federal courts (p. 424).

Lacking official statistics on self‐representation rates in Israeli courts, this practice appears relatively rare, though exact numbers are unknown. Gough and Poppe (2020) demonstrated that pro se litigation rates in U.S. federal courts remain stable over time, indicating a persistent phenomenon requiring further examination of underlying motivations. Building on these longitudinal findings, Goldschmidt (2022) analyzed how policies impact self‐representation rates, highlighting how judicial guidance, standby counsel availability, and legal aid access influence defendant decisions. His research reveals that while comprehensive legal assistance programs reduce self‐representation, overloaded and underfunded public defense services drive some defendants toward pro se status due to dissatisfaction with available representation options.

### 2.4 Why do Defendants Refuse Legal Representation and Choose to Represent Themselves?

Defendants' motivations for declining appointed counsel and choosing self‐representation are complex and multifaceted. A key driver identified in the literature is the desire to control defense strategy and decision‐making personally. Defendants often reject their public defender's proposed tactics and seek autonomy to make their own choices at trial (Hashimoto 2010, 6).

Dissatisfaction with counsel also influences decisions to self‐represent. Defendants may believe appointed lawyers are overburdened, unresponsive, or pressuring them to plead guilty rather than asserting innocence (Prescott 2010; Staudt and Hannaford 2002). As prior studies have revealed, key drivers also include distrust of the judicial system, adhesion to fringe ideologies, overconfidence in legal abilities, and personality disorders manifesting in rejection of advice (Hashimoto 2010).

Additionally, mental health factors impact some defendants' decisions. Severe mental illness can impair comprehension of the disadvantages of self‐representation (Johnston 2011). Delusions due to mental illness lead a small number of defendants to believe they possess superior legal knowledge compared to lawyers (Hashimoto 2010, 40). Further, antisocial personality traits like narcissism and paranoia are overrepresented among pro se defendants, correlating with rejection of counsel's advice.

While focusing on the Israeli context, this study builds upon prior research examining defendants' experiences with self‐representation in other jurisdictions, such as Casper's (1972) seminal work on the defendant's perspective in the U.S. and Clair (2020, 2021) recent explorations of how race and class shape defendants' interactions with the legal system and their decisions to self‐represent. By investigating these dynamics within Israel's distinct socio‐legal landscape, where ethnic and religious identity may intersect with other factors, this study contributes to a growing body of literature challenging dominant assumptions about self‐representation.

Contemporary empirical research on defendants' self‐representation experiences remains limited, with scholarship typically reflecting legal professionals' perspectives rather than defendants'. This gap is especially pronounced in Israel, where courts increasingly permit self‐representation without formally recognizing it as an absolute right. Despite Israel's comprehensive legal aid system, defendants' motivations for and experiences with waiving counsel have received minimal scholarly attention. This study addresses these gaps by providing novel empirical insights from defendants' perspectives, contributing vital understanding to an understudied area in Israeli legal scholarship.

### 2.5 The Right to Self‐Representation in Criminal Trials in Israeli Law

Self‐representation lacks explicit statutory authorization in Israel but has been established through case law. The Supreme Court first acknowledged this limited right in State of Israel v. Yolzari Ltd (2006), affirming a defendant's ability to represent himself despite court reservations. In this case, a bus driver involved in an accident causing 17 deaths initially had private counsel but later dismissed his attorney and demanded self‐representation, refusing court‐appointed defenders.

Despite initially rejecting his request due to case complexity and potential sentence severity, the Supreme Court eventually acknowledged the defendant's limited right to self‐represent after his persistent refusal to cooperate with public defenders (Public Defender's Office v. State of Israel, 2005). Following this precedent, criminal courts across all levels have increasingly recognized defendants' right to represent themselves at various procedural stages.

This recognition extends to detention hearings, criminal appeals, and extradition requests, with some defendants permitted full self‐representation and others allowed partial self‐representation with standby counsel assistance (Ilan Eshed v. The District Court in Tel Aviv, 2011; Eisenkot v. State of Israel, 2010). This evolving approach balances defendant autonomy with procedural fairness considerations (Decker 1995).

While permitting self‐representation, courts consistently note its undesirability, citing procedural complications and conviction risks, yet acknowledge that forced representation would violate defendant autonomy—an interest that ultimately prevails (Kennedy v. State of Israel, 2009; State of Israel v. Vinner, 2015; State of Israel v. Barsky, 2014). Judges must guide pro se defendants without encouraging self‐incrimination or interfering excessively, and must appoint standby counsel when needed (Farber 2020; Assy 2024).

For defendants with suspected mental health conditions affecting competency, courts may order psychiatric evaluations and conduct competency hearings where representation typically cannot be refused. If deemed incompetent, defendants receive compulsory representation, highlighting the tension between protection and autonomy central to self‐representation discussions. Understanding motivations for exercising this right requires examining defendants' direct experiences.

## 3 Methodology

The research aimed to explore the experiences of adult unrepresented defendants in the Israeli criminal courts, examine why defendants were self‐represented, and identify the resources they used. In other words, the research question was: Why do criminal defendants in Israel waive their right to legal counsel and choose to self‐represent, and what challenges do they face during this process? This study explored why defendants in criminal cases waived their right to legal representation provided by the state (via the public defender's office) in Israel. The study adopted a qualitative approach, using semi‐structured interviews as the primary data collection method, accompanied by courtroom observations during such proceedings.

### 3.1 Sampling

The study employed purposive sampling to interview 16 participants who were criminal defendants who waived their right to legal representation and chose to self‐represent in court. Among the participants, 9 (56.25%) were Jewish, 4 (25%) were Muslim, and 3 (18.75%) were Christian or had no religious affiliation. The age distribution was as follows: 2 (12.5%) were 30–40 years old, 8 (50%) were 40–50 years old, 2 (12.5%) were 50–60 years old, and 4 (25%) were 60–70 years old. The majority of the participants (14, or 87.5%) were male, with only 2 (12.5%) being female.

Regarding income, 10 (62.5%) of the interviewees reported earning above the minimum wage in Israel (approximately $1600) but less than the average wage (approximately $3600), while 4 (25%) reported earning more than the average wage. Two participants (12.5%) declined to answer the income question. The participants were involved in various criminal proceedings across all levels of the Israeli judicial system, including the Supreme Court, district courts, and magistrate courts throughout northern, central, and southern Israel. These proceedings encompassed a range of legal matters such as trials, appeals, detention hearings, and other criminal procedures. Table 1 presents the characteristics of the study participants.

**TABLE 1:** Summary of participants by age, gender, income, and religion.

Characteristic	Subcategory	Percentage (%)
Age	30–40	13
	40–50	50
	50–60	13
	60–70	25
Gender	Female	12
	Male	88
Income	Above average	25
	Above minimum, below average	63
	Declined	12
Religion	Christian/None	19
	Jewish	56
	Muslim	25

The offenses filed against the interviewees included homicide/manslaughter (1 participant), sexual crimes (2 participants), violent offenses (9 participants), and economic offenses (4 participants). More than half of the 16 interviewees had previous experiences with the criminal justice system, such as prior arrests and prosecutions within a criminal trial. In addition to the personal interviews, publicly available judgments or judicial decisions regarding some of the interviewees were also used from a legal database known as “Nevo.”

The sample size was relatively small due to the difficulty of finding and accessing participants who fit the criteria. This difficulty was partly related to the fact that the right to self‐representation in Israel was only semi‐officially recognized a few years before the study was conducted, leading to a limited number of cases in which defendants exercised this right. Additionally, the State of Israel provides substantial legal assistance to individuals facing allegations of serious crimes (subject to economic criteria), which may mitigate the occurrence of self‐representation in criminal cases. Moreover, some potential participants were reluctant or unwilling to participate in the study due to their cases' sensitive and personal nature.

Participants were recruited through court records (*n* = 8), online forum postings (*n* = 5), and personal referrals (*n* = 3). Eligibility criteria included adult defendants involved in criminal proceedings requiring legal representation under Israeli law, who chose to self‐represent at some stage. Self‐representation in this study extended beyond full criminal trials to various legal proceedings, including extradition hearings, criminal appeals, and detention proceedings. Interviews were conducted at different stages of the legal process, with most (*n* = 12) occurring during trial, while others took place in the context of appeals, extradition requests, or detention hearings—highlighting the varied contexts in which self‐representation occurs.

For the purposes of this study, 'self‐representation' was observed to encompass a range of scenarios rather than a single, uniform phenomenon. In the Israeli cases examined, this included not only defendants who formally represented themselves throughout their entire proceedings but also those who navigated various intermediate arrangements specific to Israeli courts. Analysis of the cases in this research led to the identification of five main types of self‐representation in Israel:

1. Selective self‐representation: Defendants who chose to represent themselves only in certain parts of the proceedings, such as during the evidentiary stage or in *specific* hearings of the evidence phase. For example, one interviewee was represented during the trial stage but represented himself during the appeal.
2. Mixed self‐representation: Situations where the defendant and defense counsel shared representation duties, with the defendant managing certain aspects of the defense (such as cross‐examining) while the counsel handled other aspects, sometimes in agreement and sometimes amid tension between the parties.
3. Involuntary self‐representation: Cases where defendants whose requests to replace counsel were denied refused to cooperate with their appointed counsel, leading to a practical situation of partial self‐representation, despite formally still being represented.
4. Temporary self‐representation: Defendants who represented themselves for interim periods, sometimes lengthy ones, such as while the court considered a request to release their attorney or during transitions between attorneys.
5. “Ambiguous” self‐representation: Situations where the representation status remained formally ambiguous despite the defendant effectively managing their defense independently.

This spectrum of self‐representation was evident in several cases observed during the research. For example, one interviewee began with legal representation, dismissed his attorney and continued to represent himself for several months, but shortly before the ruling on his extradition proceedings, requested legal representation again. Another interviewee dismissed his attorney and chose to represent himself, which created prolonged delays in the trial due to the disconnect between him and his appointed counsel. Finally, after a long period of procedural complications, the court allowed the defendant to perform certain legal actions ‐ without issuing a formal decision to release the counsel.

These variations highlight that self‐representation in Israel's criminal justice system is not a binary status but rather exists on a spectrum, influenced by judicial discretion, procedural developments, and defendants' changing circumstances. This complexity emphasizes the need to understand self‐representation not merely as an expression of autonomy but as a flexible and context‐dependent process shaped by both institutional constraints and personal choices.

In addition to the interviews, 22 observations of court hearings were conducted regarding some of the self‐represented defendants who were interviewed. These observations provided additional context on the challenges and strategies employed by self‐represented litigants in practice, allowing for a more comprehensive understanding of the phenomenon beyond individual narratives.

### 3.2 Data Collection

The data collection took place between January and August 2023. Participants were sourced through court records, online forums, and personal referrals. The study's purpose and procedures were explained, informed consent was obtained, and interviews were scheduled. The interviews were primarily conducted via Zoom, except for two participants who preferred to meet in person and one interview that was conducted by telephone. The interviews lasted approximately 30 min to an hour and followed a semi‐structured format with open‐ended questions that allowed participants to express their views and experiences in their own words.

The interview protocol explored several key areas related to self‐representation experiences. These included: the participants' legal case details and their rationale for declining free legal assistance; their perceptions of the advantages and disadvantages of self‐representation; the specific challenges and difficulties they encountered while representing themselves in court; the outcomes of their cases and the personal significance of their self‐representation experience; and their levels of satisfaction or regret regarding their decision to represent themselves. This comprehensive approach allowed for a holistic understanding of participants' experiences with the criminal justice system when navigating it without professional legal counsel.

The interview protocol was developed by consulting Tyler's (2003) model of procedural justice(Casper et al. 1988), which emphasizes individuals' perceptions of fairness in their interactions with legal authorities. Key elements like voice, respect, trustworthiness, and neutrality were incorporated into the interview questions to understand participants' overall satisfaction with the legal process. However, to minimize potential bias and avoid leading respondents (Hollander‐Blumoff and Tyler 2008), these themes were explored through open‐ended questions (e.g., “Can you describe your experiences in court?'” rather than direct or suggestive phrasing (e.g., “Did you feel treated fairly?”)

### 3.3 Data Analysis

Analysis followed Braun and Clarke's (2006) thematic analysis method, comprising six steps: data familiarization through repeated reading, initial coding, theme identification, theme review and refinement, theme definition and naming, and final analysis with supporting extracts. Analysis validation employed four strategies: triangulation across multiple data sources and participants, member checking of preliminary findings, peer debriefing with topic and methodology experts, and reflexive practices addressing potential researcher biases. For reporting theme prevalence in the Findings section, specific thresholds were established: “some” indicates 1–5 participants (of 16 total) expressed a view, while 'most' indicates six or more shared perspectives, providing clarity on perspective commonality within the sample.

## 4 Findings

Before presenting the findings, it is important to clarify that self‐representation in the Israeli legal system, as observed in this study, varied in degree rather than constituting a uniform state of complete legal independence. Some defendants represented themselves throughout the proceedings, while others navigated transitional phases as courts deliberated on their requests to dismiss counsel. In some cases, courts granted partial autonomy without formally releasing attorneys, reflecting a unique practice within the Israeli system. These variations indicate that self‐representation in Israel operates on a spectrum, with legal autonomy often negotiated rather than fully granted or denied.

Participants often cited multiple, intersecting reasons for choosing to represent themselves. The most commonly reported motivations were a desire for personal involvement in the legal process (cited by 9 out of 16 participants), a lack of trust in legal counsel (cited by 7 out of 16 participants), and mental health issues (cited by 4 participants). Other influential factors mentioned by some participants included underestimation of court system complexity (cited by 3 participants), dissatisfaction with prior legal outcomes (cited by 2 participants), and dissatisfaction with attorney advice (cited by 2 participants).

Within this complex landscape of varying representational arrangements, participants' motivations for seeking self‐representation proved equally multifaceted. While this study did not systematically examine how socioeconomic or religious backgrounds influence self‐representation, several participants alluded to a broader sense of distrust toward the legal system. For instance, one participant stated: “The lawyer they assigned me barely listened to my side of the story. I knew from the beginning that they saw me as just another case to clear.” Another remarked: “I have been through this system before, and I know that the public defenders don't really fight for people like me.” These sentiments echo findings in previous research suggesting that perceived bias in legal institutions can contribute to defendants' reluctance to engage with court‐appointed attorneys (Clair 2021; Kohler‐Hausmann 2018).

However, due to the study's methodological limitations, it is impossible to draw definitive conclusions about whether factors such as religion or socioeconomic status played a decisive role in participants' self‐representations. Future research incorporating a broader dataset and direct questions about these variables could provide more clarity on this issue.

### 4.1 Mental Health Issues

One of the themes that emerged from the interviews was the impact of mental health issues on the decision to waive legal representation. Some of the participants reported having mental health problems such as depression, anxiety, or post‐traumatic stress disorder (PTSD) that affected their ability to trust others, communicate effectively, or cope with stress. For example, one participant said: “I have PTSD from my time in the army when I got Injured. I like to be quiet. I do not like having many people around me, especially if they are loud, like lawyers. I cannot trust them. I can only trust myself. That is why I decided to represent myself.” (Participant C, January 21, 2023).

Another participant noted that: “I have struggled with anxiety and panic attacks since I was a teenager. I do not remember exactly when it started, but it has been with me for most of my life. If I take the pills ‐ everything is fine…The thought of handing my case over to a lawyer and not being in control was terrifying. Whenever I tried to explain things to my attorney, I would get so anxious that I could not get the words out right. I was paranoid that they would twist my story…" (Participant K, February 5, 2023).

Participant M also added, “I suffer from obsessive‐compulsive disorder (OCD), which causes intrusive thoughts and intense anxiety. When I first got arrested, the public defender assigned to me seemed very impatient and dismissive. She was not interested in hearing about all the details that mattered so much to me. I felt anxious about her handling my case…”. (Participant M, May 16, 2023). Participant L further illustrated how mental health challenges influenced the decision to self‐represent: “My disorder makes it difficult for me to open up to people, even lawyers who are supposed to be on my side. The paranoia made self‐representation feel like the only option where I could be fully in control.” (Participant L, April 3, 2023).

### 4.2 Desire for Personal Involvement

Another theme that emerged from the interviews was the desire for personal involvement in the legal process. Some participants expressed a strong sense of ownership and responsibility for their case and wanted more control and autonomy over their defense. For example, one participant said: “I wanted to be involved in every aspect of my case. I wanted to know what was happening, the evidence, and the arguments. I wanted to have a say in everything. I did not want to leave it to someone else who does not care or does not understand. I felt like I owed it to myself and to the truth.” (Participant D, January 21, 2023).

Another participant noted, “I could not just sit there silently while my lawyer spoke for me. This was my life on the line. I wanted to be active, not just an observer. I wanted the judge to hear my voice directly from me. My lawyer did not understand this case as deeply and emotionally as I did….” (Participant J, January 30, 2023). Moreover, “No one could convey my experiences' depth and complexity like me.” Moreover, “When my fate is being decided, I will not just sit mutely while someone else speaks for me. My whole future depends on the judge connecting with the truth as I convey it straight from my mouth and heart.” (Participant G, April 25, 2023). Participant N expressed a similar motivation for self‐representation: “I have been through the criminal justice system before, and I always felt like a passive bystander while my lawyer controlled everything. This time, I needed to take an active role and make my own choices.”

Courtroom observations provided further insight into defendants' practical experiences. Judges varied significantly in the degree of assistance provided to self‐represented defendants. Some judges actively guided defendants, suggested appropriate questions, and even posed clarifying questions themselves, attempting to support defendants and partially level the playing field. In other instances, judges maintained a more passive role to preserve judicial neutrality. This variation underscores the need for clearer guidelines regarding judicial roles and responsibilities (Rosenbloom 2002) toward self‐represented defendants in Israeli courts.

### 4.3 Underestimation of Court System Complexity

A third theme that emerged from the interviews was an underestimation of the legal system's complexity. Some of the participants admitted that they did not fully appreciate the challenges and difficulties of representing themselves in court. They underestimated the amount of work, knowledge, and skills required to represent themselves. They also underestimated the power and influence of the prosecution and the judge. For example, one participant said: “I thought it would be easy. I did not realize how complicated the legal system was. There were so many rules and procedures that I found out during the proceedings. There were so many documents and motions that I had to file.” (Participant E, January 21, 2023).

Moreover, “I figured the judge would just let me explain my side. I had no idea about all the rules of evidence and procedure. When the prosecutor made motions to exclude information I wanted to present, I was caught completely off guard. I was overwhelmed trying to understand legal jargon and make persuasive counter‐arguments.” (Participant A, February 10, 2023). Another participant added, “I assumed I could just go and tell it to the judge straight. I did not know there would be all these loopholes on what you can and cannot say”. (Participant F, January 21, 2023). Participant O shared his surprise at the procedural requirements: “I thought It would be easy. I was wrong.” (Participant O, April 12, 2023).

### 4.4 Dissatisfaction With Prior Legal Outcomes

A fourth theme that emerged from the interviews was dissatisfaction with prior legal outcomes. Some of the participants had previous experiences with the legal system that resulted in damaging or unsatisfactory outcomes. They felt they were mistreated, wrongly, or given harsh sentences. They blamed their lawyers for their poor performance or incompetence. They lost faith in the legal system and decided to take matters into their own hands. For example, one participant said: “I had a bad experience with a lawyer before. He was a public defender who did not care about my case. He did not listen to me or investigate my case. He pressured me to take a plea deal that I did not want to take… I vowed never to trust a lawyer again. I decided to represent myself this time. I could do a better job than him.” (Participant F, January 21, 2023).

Participant B added, “My previous lawyer really let me down… (“He f***ed me twice”) Because of his lazy defense and his other clients, I spent months in jail. I spoke with a lawyer they gave me, but he did not believe me. He treated me like I was guilty. I realized I could not rely on the legal system. My only hope was taking charge of my own defense, so at least I would have a fighting chance to tell my side of the story…" (Participant B, February 12, 2023).

### 4.5 Dissatisfaction With Attorney Advice

A fifth theme that emerged from the interviews was dissatisfaction with attorney advice. Some participants consulted with attorneys before deciding to waive legal representation. They were dissatisfied with the advice they received from their attorneys. They felt their attorneys did not understand their situation. They disagreed with their attorneys' strategies, recommendations, or opinions. For example, one participant said: “I talked to a lawyer before I went to court. He told me to accept the deal. He said it was the best option for me. He said I had no chance of winning at trial. He said the evidence against me was too strong. He said I would get a longer sentence if I went to trial. I did not want to admit to something I did not do. I wanted to fight for my innocence. I decided to represent myself.” (Participant G, April 25, 2023).

Another participant stated, “The lawyer I consulted told me that the prosecution's case was solid and that I would likely lose if I chose to go to trial. I disagreed with him. I felt he did not fully grasp the nuances of my case to prove my innocence. I did not want to plead guilty. So, I rejected his advice and represented myself.” This participant expressed that, “From the beginning, I felt my lawyer was not aggressive enough in representing me. When I described the situation, I expected a more forceful approach. However, my lawyer kept advocating for a conciliatory resolution, emphasizing the risks and costs of a lengthy legal battle.” (Participant A, February 10, 2023).

### 4.6 Lack of Trust in Legal Counsel

A sixth theme that emerged from the interviews was the lack of trust in legal counsel. Some of the participants expressed a general distrust or suspicion of lawyers and the legal profession. They sometimes believed that lawyers were unprofessional, dishonest, greedy, or manipulative. They feared that lawyers would exploit them or harm them. They preferred to rely on themselves rather than on lawyers. For example, one participant said: “I do not trust lawyers. They are all liars. They do not care about their clients. They will say one thing and do another. They will make promises and break them. They will stab you in the back. I do not need a lawyer.” (Participant H, May 11, 2023).

Participant I added, “The public defender assigned to me barely looked at my case file. During our first meeting, he kept checking his phone. It was clear he saw me as just another number. I had no faith that he would fight for me. I could not entrust my future to someone who did not care.” (Participant I, May 23, 2023). Moreover, “finally, they gave me a lawyer with the face of a high school boy. My grandson looks older than him. I asked them for a replacement, but they disagreed”. This participant added that, “Honestly, I did not feel comfortable trusting my lawyer because of the communication gap we had.” (Participant J, January 30, 2023).

## 5 Discussion

This study provides a nuanced and empirically grounded examination of self‐represented defendants in Israel's criminal justice system, shedding light on the motivations, challenges, and broader implications of this legal phenomenon. While self‐representation is often framed in legal discourse as an exercise of autonomy, the findings illustrate that the decision to forgo legal counsel is rarely a purely voluntary or strategic choice. Instead, it is shaped by intersecting psychological, structural, and experiential factors that complicate conventional understandings of defendant agency.

### 5.1 Key Findings and Their Implications

The first research question—*What motivates defendants to self‐represent rather than use free legal services?* revealed several primary drivers of this decision. Mental health challenges, such as PTSD, anxiety, and OCD, played a significant role in limiting defendants' ability to trust and communicate effectively with legal counsel, echoing prior studies that have linked psychiatric conditions to self‐representation (Hashimoto 2010; Johnston 2011). A strong desire for direct control over one's defense was another major factor, with participants expressing a sense of ownership over their cases and deep skepticism toward legal representatives.

Additionally, many defendants underestimated the complexities of the legal system, leading them to believe they could effectively advocate for themselves despite lacking legal training (Poppe and Rachlinski 2015). Prior dissatisfaction with legal representation—either due to perceived incompetence, excessive caseloads, or a lack of advocacy—further contributed to the decision to self‐represent, reinforcing broader concerns about trust in the public defense system (Toy‐Cronin 2017; Goldschmidt 2022).

In terms of case outcomes, the small sample size prevents drawing definitive conclusions regarding the relationship between self‐representation and case disposition. Future studies with larger, more representative samples could clarify these dynamics further.

The second research question—*What difficulties do self‐represented defendants face?* —highlighted procedural, evidentiary, and strategic challenges. Participants described struggling to navigate courtroom protocols, effectively cross‐examine witnesses, and introduce admissible evidence. These findings align with longstanding critiques of self‐representation, which argue that defendants who lack legal training are at a significant disadvantage and may inadvertently undermine their own cases (Quintanilla et al. 2017; Engler 1998; Buhai 2008). The findings underscore the need for additional safeguards to prevent self‐representation from becoming an exercise in systemic self‐sabotage.

### 5.2 Theoretical Contributions and the Role of Structural Inequality

Beyond procedural concerns, this study contributes to the broader scholarly discourse on inequality in the legal system. While self‐representation is often framed as an individual choice, these findings suggest it frequently reflects structural disenfranchisement. Building on Clair's (2021) and Kohler‐Hausmann's (2018) work, this study highlights how negative experiences with public defenders, institutional distrust, and economic precarity push some defendants toward self‐representation despite its risks.

Additionally, systemic constraints within Israel's public defense system—such as chronic underfunding and high caseloads—may further pressure defendants into self‐representation. Limited resources often lead attorneys to prioritize plea bargains over vigorous defense, leaving some defendants feeling underserved. Future studies should further examine how resource limitations impact defendants' decisions to waive counsel. However, marginalization was not the primary motivation for all participants; many cited PTSD, misjudgment of legal complexity, or a desire for control, underscoring the need for a nuanced interpretation.

From a Foucauldian perspective, self‐representation can be seen as both an assertion of agency and a symptom of systemic exclusion. As Foucault (1982) argues, legal institutions shape behavior under the guise of neutrality. While defendants may perceive self‐representation as reclaiming control, they often face structural disadvantages—challenging the assumption that self‐representation is purely an exercise of rights rather than an indicator of systemic failure.

### 5.3 Limitations and Directions for Future Research

Despite its significant contributions, this study has several limitations. First, the small, non‐random sample of 16 participants limits the generalizability of the findings. Although qualitative research prioritizes depth over breadth, future studies should include larger and more diverse samples to allow comparative analyses across various legal, demographic, and socio‐cultural contexts.

Second, relying on semi‐structured interviews may introduce biases such as retrospective framing, recall, or social desirability bias. While member checking and peer debriefing were used to mitigate these issues, future research could benefit from triangulating interview data with additional sources like court records or psychological assessments.

Third, the study's cross‐sectional design—interviewing participants at a single point (post‐indictment but pre‐case resolution)—does not capture how perceptions evolve over time. A longitudinal design with interviews at multiple stages (e.g., post‐indictment, during trial, and post‐sentencing) would provide a more dynamic understanding of self‐representation.

Finally, although social factors like income and religion were mentioned, their intersection with self‐representation decisions was not comprehensively analyzed. Future work should explore whether certain groups are disproportionately inclined to self‐represent and if systemic biases contribute to this trend. By acknowledging these limitations—a small, non‐random sample, potential biases in self‐reporting, a cross‐sectional design, and limited socio‐cultural analysis—this study sets the stage for further research to refine and expand upon these findings.

### 5.4 Rethinking Self‐Representation in Criminal Justice

By centering the voices of self‐represented defendants, this study challenges dominant legal narratives that view self‐representation as either an unproblematic exercise of rights or a procedural nuisance. Instead, it reveals self‐representation as a deeply complex phenomenon—one that reflects both individual agency and systemic constraints. The findings call for a more nuanced policy approach that balances the right to self‐representation with meaningful safeguards, such as improved public defense services, enhanced legal education, and tailored support systems for vulnerable defendants.

The legal system, at its core, is meant to ensure fairness, yet self‐representation—when exercised under conditions of disadvantages, which raise fundamental concerns about access to justice. As this study demonstrates, self‐representation is not always an empowered choice but often a constrained one. Moving forward, legal scholars and policymakers must grapple with this reality, ensuring that defendants who waive counsel do so with informed agency rather than out of systemic necessity.

Ultimately, this research contributes to an ongoing, urgent conversation about legal autonomy, procedural justice, and the broader implications of self‐representation in modern criminal justice systems. By illuminating the lived experiences of self‐represented defendants in Israel, this study lays the groundwork for more inclusive, empirically informed discussions on the future of legal representation worldwide.

#### 5.4.1 Policy Implications and Recommendations

This study's empirical findings inform several targeted policy recommendations designed to address the challenges identified in self‐representation cases while respecting defendant autonomy. The following recommendations directly respond to participants' experiences and are calibrated to the strength of supporting evidence from this research.

### 5.5 Self‐Representation Support Framework

The research reveals that the desire for personal involvement in the legal process was the predominant motivation for self‐representation, cited by 9 of 16 participants. As Participant D articulated: “I wanted to be involved in every aspect of my case… I wanted to have a say in everything.” This finding provides a strong empirical foundation for enhancing the existing standby counsel system in Israel's courts.

A structured hybrid representation model that provides proactive legal guidance at critical trial stages without diminishing defendant agency is proposed. Drawing on Participatory Defense principles (Godsoe 2018) but adapted for institutional implementation within Israel's legal system, this approach would establish scheduled consultations with standby counsel during pretrial motions, evidentiary hearings, and closing arguments. This balanced approach directly addresses both participants' expressed need for control and the procedural difficulties observed during court observations. While enhancing support for those who choose self‐representation addresses one dimension of the findings, equally important is addressing the underlying factors driving this decision.

#### 5.5.1 Public Defense Enhancement

The substantial trust deficit between defendants and public defenders emerged as a significant finding, with 7 participants explicitly citing mistrust as a factor in their decision to self‐represent. Participant I's experience exemplifies this issue: “The public defender assigned to me barely looked at my case file… It was clear he saw me as just another number.”

To address this documented trust deficit, implementation of a systematic client feedback mechanism within Israel's public defense system is recommended. This approach could adapt frameworks such as the Structured Shadow Method (Campbell and Henderson 2022), which evaluates attorney‐client communication and identifies opportunities for improvement. Specifically, structured feedback should be systematically collected through brief questionnaires or interviews conducted by an independent supervisor after initial client‐attorney meetings, significant pre‐trial hearings, and at the conclusion of trial, enabling attorneys to adjust their methods continuously to align with defendants' needs and expectations. Additionally, the findings regarding defendants' perceptions of attorney disengagement suggest the need for enhanced training in client‐centered advocacy and communication skills.

#### 5.5.2 Additional Support Measures

Mental health challenges influenced self‐representation decisions for some participants. Four defendants described how conditions such as PTSD, anxiety, and OCD affected their ability to work with legal counsel. While this represents a smaller proportion of the sample, these accounts align with previous research on the relationship between mental health and legal decision‐making (Johnston 2011). Based on these findings, optional psychological support resources should be developed for defendants considering self‐representation. Rather than mandatory screening, which could raise concerns about autonomy, this approach would make consultation services available to defendants who might benefit from them. Given the limited empirical support from the study for this recommendation, pilot implementation and further research would be appropriate before broader adoption.

#### 5.5.3 Implementation Considerations

The effectiveness of these recommendations depends on implementation that recognizes the complex motivations for self‐representation identified in this study. Institutional change requires substantial resources and coordination. Therefore, enhancing standby counsel systems should be prioritized, as this approach has the strongest empirical foundation and addresses defendants' commonly expressed desire for greater involvement in their cases. Additionally, developing systems to track self‐representation outcomes would generate valuable data for refining these interventions.

By monitoring both case results and defendant experiences, courts can develop policies based on empirical evidence rather than assumptions about self‐represented defendants' needs. Given participants' strong desire for direct involvement, the Israeli system might consider adopting practices from European jurisdictions that allow unsworn testimony, enabling defendants to actively participate without fully waiving legal representation, thereby providing greater voice while reducing the perceived need for complete self‐representation.

## 6 Conclusion

This study examined the motivations and experiences of Israeli criminal defendants who waived free legal counsel for self‐representation. Rather than following a binary model, self‐representation in Israel exists on a spectrum from full independence to partial autonomy arrangements. Through interviews with 16 defendants and courtroom observations, the research identified driving factors including mental health challenges, desire for control, underestimation of legal complexities, dissatisfaction with legal services, and distrust of attorneys.

By centering defendants' narratives, this research contributes empirical insights into an understudied phenomenon, revealing psychological and emotional dimensions behind self‐representation and elevating perspectives often marginalized in policy discussions.

Despite these contributions, limitations exist: the small, non‐random sample limits generalizability, while the cross‐sectional design fails to capture evolving perceptions. Future research should adopt longitudinal approaches, incorporate larger samples, integrate multiple data sources, and explicitly examine defendants' experiences with standby counsel—an aspect not fully explored in this study despite Hashimoto's (2010) findings that pro se defendants often perceive standby counsel as either intrusive or insufficient. Ideally, future studies would include matched comparison groups of represented defendants, though practical constraints prevented this approach here.

The findings suggest several policy implications: expanding standby counsel models to balance autonomy with guidance, strengthening public defender communication through training and oversight, and implementing mental health screening during waiver assessments.

Ultimately, this study highlights the tension between legal autonomy and procedural fairness. While framed as a right, self‐representation often reflects systemic failures rather than informed choice, requiring nuanced, evidence‐based approaches that protect both defendant agency and judicial integrity.

## Author Contributions

The author was solely responsible for the conceptualization, methodology, data collection, analysis, writing, and editing of this manuscript.

## Ethics Statement

This study adhered to accepted ethical guidelines for research with human subjects. All participants provided informed consent after receiving full information about the study's purpose and confidentiality measures. Participant identities were protected through anonymization of all data.

## Conflicts of Interest

The author declares no conflicts of interest.

## Data Availability

The interview data generated during the current study are not publicly available due to confidentiality but are available from the author upon reasonable request and subject to ethical approval.
